# Wound healing center establishment and new technology application in improving the wound healing quality in China

**DOI:** 10.1093/burnst/tkaa038

**Published:** 2020-10-26

**Authors:** Xiaobing Fu

**Affiliations:** Wound Healing Unit, Medical Innovation Department and the Fourth Medical Center, PLA General Hospital and PLA Medical College, 28 Fu Xian Road, Haidian District, Beijing 100853, China

**Keywords:** Trauma, Wound healing, Tissue repair, Wound healing center

## Abstract

Wound healing, tissue repair and regenerative medicine are in great demand, and great achievements in these fields have been made. In recent years, many of these successes have benefitted patients, especially in the field of chronic skin wounds. However, perfect tissue repair and regeneration of damaged tissues and organs are still great challenges in the management of trauma and diseases. In this paper, the main achievements in wound healing, tissue repair and regeneration in China are reviewed and the establishment of wound healing centers and new technology application in improving wound healing quality in patients in China is highlighted.

## Wound care is a heavy burden in China for all Society

According to previous studies, about 100 million patients need wound care every year in China, and about 30 million of these have severe skin wounds. Wound care is a very heavy burden, not just for the patient’s family but for all society. Our data showed that one patient with severe traumatic skin wounds, diabetic foot or venous ulcers usually needs about two more individuals to look after him/her, and, in 2008, the average length of stay was 21 days, compared with 8.6 days in hospital for other patients. Also, there is a very heavy medical cost for these patients. In 2008, the average cost for managing chronic skin ulcers care in one patient was 12,227 RMB Yuan (about $1,772 USD); however, the cost of other diseases was only 4,132 RMB Yuan (about $599 USD) [1]. In 2018, the average cost of hospitalization for chronic skin ulcers was 55,270 RMB Yuan per capita (about $8,010 USD). Thus, management of these patients with severe skin ulcers is a great challenge not just for doctors and nurses but also for government administrators,etc.

## Invention of wound management systems is key

To meet these great challenges, an invention system of wound care management should be established, and advanced technologies should be used. In recent years, the practices in establishing a new model to prevent and treat these hard-healing skin wounds have been developed, with successful results.

Three epidemiological studies were completed in 1998, 2008 and 2018, and some of the results were published. We have discovered new epidemiological characteristics of chronic skin ulcers in China. The main causes of chronic skin ulcers in China changed from trauma to diabetic foot ulcers from 1998 to 2018. In 1998, the main causes of chronic skin ulcers were trauma and infections, which accounted for about 67.5% of all chronic skin ulcers, whilst only 4.9% were due to diabetic foot. However, just 10 years later, chronic skin ulcers caused by trauma and infections reduced to 24%, and ulcers caused by diabetic foot increased to 33%. In 2018, a new epidemiological study showed that the first cause of chronic skin ulcers was still diabetic foot, representing about 25.3% of all chronic skin ulcers. Thus, the prevention and management strategy should be changed from a “traumatic model” to a “disease model” to reflect differences in pathophysiology and management methods between traumatic skin wounds and skin wound-induced diseases, such as diabetic foot. These basic data support the epidemiological variation trend of diabetes mellitus in China: some data show that the incidence of diabetes mellitus increased from 2.5% in 1994 to 11.6% in 2013. In recent years, the Chinese government has paid much attention to the prevention of diabetes mellitus and research funds for its study were increased [1, 2, 3].

Professional training is helpful in improving the abilities of doctors and nurses to manage chronic skin wounds [4]. Therefore, training programs for chronic skin wound management, such as diabetic foot prevention, were established and guidance for diagnosis and treatment of chronic wounds was promoted. These were funded by the World Diabetic Foundation and the Coloplast Access to Healthcare Partnership Program in 2010. The aim of this program was to constantly promote the standardization of treatment of diabetic foot and relative chronic skin wounds in China [5]. By the end of 2015, 9229 doctors or nurses had been trained and 60 workshops were held; about 4900 diabetic patients benefitted from improved wound healing time. By the end of 2015, wound care projects were launched in 42 hospitals with the support of this training program. After that, the Branch of Trauma Surgeons of the Chinese Medical Association continued to perform training for wound care doctors and nurses and the results are very satisfying.

Doctors and hospital managers continuously face the issue of how to treat these chronic skin ulcers in the hospital ward or outpatient care. In the past years, no hospital had a specialized wound repair department, and wound care doctors or techniques were limited in hospitals. The nurses were the main force in treating skin wounds in hospital wards or in outpatient care. Their main tasks for wound care were the changing of dressings. However, if chronic skin wounds were treated by doctors, these doctors were not professional wound care doctors, and they usually came from different departments, such as burn units, general surgery, orthopedics and endocrinology; their knowledge and skills could not meet the standard of professional therapy required for these complicated cases, and only about 60% of skin wounds healed when patients were discharged from the hospital.

## Establishing special wound care units or centers in hospitals is a successful model

Thus, it is necessary to establish a new model for complicated skin wound treatment. Since 2010, China has begun establishing special wound care units or centers in hospitals. The wound care units or centers have the following characteristics: all skin wounds are considered complicated diseases caused by trauma or diseases; and all doctors or nurses treating these wounds are trained in professional wound care courses before becoming wound care specialists. Also, multidisciplinary approaches were used and connections with community medical units are established. This new model helps to reduce the number of hospitalization days for patients when they complete intensive management, such as surgery and skin transplantation in hospital and then are moved to the community medical unit for future rehabilitation and treatment.

The number of wound care units or centers increased from 16 before 2010 to 357 in 2019. These wound care units or centers are located in all geographic areas, but mainly in eastern China. Recently, studies in 69 wound healing units or centers showed that the number of patients who visited the wound care units per month increased from 127 to 984, the average hospital stay of these patients decreased from 21 days to 16 days, the rate of wound healing increased from 47% to 87% and the time it took wounds to heal decreased from 114 to 69 days. The scope of treatment in wound care units or centers was enlarged and some very complicated chronic skin wounds, such as leg sheet wounds present for 68 years and eclectic foot ulcers present for 52 years, were healed [5–8]. In December 2019, the National Health Commission of China released a notice on promoting the management of the diagnosis and treatment of chronic skin wounds, which offers a chance to reform China’s prevention and treatment system for chronic skin ulcers. This system includes setting up a wound treatment specialty department, training specialized doctors and nurses and establishing a linkage of community and hospitals for wound care. Thus, the state edict offers a chance to appropriately treat chronic skin ulcers in China [8].

## Advanced techniques enhance wound healing speed and quality

The new techniques and their application are important in enhancing wound healing speed and quality [9, 10]. In the past, traditional medicine and dressings were the main treatment used for managing skin injury, but results were limited. New concepts emphasize that the wound healing speed and quality could be accelerated and improved by using advanced techniques and new methods. The new dressings play a key role not just in covering the wounds, but also in accelerating wound healing [11, 12]. In some new wound care units or centers in China, some comprehensive methods were used to treat different kinds of chronic skin wounds. One of the innovation methods in this field was the use of recombinant growth factors with genetic engineering. These growth factors include epidermal growth factors and fibroblast growth factors [13]. The results showed that wound healing time was shortened by about 2 to 4 days for acute skin wounds, such as superficial second-degree burns and donor sites. The wound healing rate was increased by 10% for chronic skin ulcers. Biomaterials, such as collagen and artificial dermal materials, are also used in managing chronic skin ulcers [14, 15]. These biomaterials help in reconstructing the dermis after deep and extensive debridement in skin wounds, especially for severe infection skin wounds, diabetic foot (over Wagner classification 3) and radiating chronic skin wounds. Negative-pressure wound therapy and new advanced dressings play a key role in wound bed preparation and establishment of wound healing microenvironments. The ow-level laser therapy plays a key role in reducing glycosylation products in the diabetic foot and enhancing DNA synthesis in repair cells, such as epidermal cells and fibroblasts, for healed wounds. Blood vessel regeneration with bone-marrow mesenchymal stem cells therapy in lower-extremity wounds, including diabetic foot, were very successful in about 2000 cases, and the effective rate was about 85% [16].

With the establishment of new wound care systems and the use of advanced techniques in managing chronic skin wounds in recent years, the Chinese Tissue Repair Society has developed a program to provide medical aid for chronic skin wounds in patients living in Zhejiang Province in eastern China. Some of these patients have suffered severe leg ulcers for at least 50 to 70 years, since World War II. As a medical association, the Chinese Tissue Repair Society set up a program to help with healing, or at least improving quality of life. The first 12 patients were sent to Shanghai and 8 were admitted to the Wound Healing Unit at the Ninth People’s Hospital affiliated to Shanghai Jiao Tong University School of Medicine. The average age of the 8 patients was about 89 years and the history of chronic skin wounds was more than 70 years. After careful assessment, a therapeutic plan was developed. The wound sites were located in the lower leg or about the stocking area. Because of the long history, the morphological characteristics of these skin wounds included fibrosis and calcification over the wound bed. Bacteriological examination showed some conditional pathogens but not *bacillus anthracis*. Before admission in Shanghai, some of these patients had visited the local hospitals many times for the management of their skin wounds, but the treatments failed. In the Wound Healing Unit at the Ninth People’s Hospital in Shanghai, after finishing the assessment, the key issue in beginning to repair the skin wounds was to cut off the excess fibrosis and calcification. All patients received standard treatment. Briefly, fibrosis and calcification tissue were removed by surgical debridement. After this, negative-pressure wound therapy was used in the wound bed for 1 or 2 weeks depending on the assessment of the wound bed condition. When the wound bed preparation was finished, skin grafting was performed. This standard therapy procedure was aimed at getting rid of the core factor of impaired issue and differed from methods used before, but ensured successful treatment. After discharge from the hospital, the wound rehabilitation plan was implemented with help from the local community healthcare system to ensure that the patients were cared for. To ensure the quality of wound care in the community healthcare system, a telemedicine system was engaged for real-time supervision by hospital-based experts in Shanghai [17]. Following these successful experiences, a new attempt was made in Zhejiang Province to help patients with chronic skin wounds, as there are still more than 700 older patients who need such medical support as soon as possible in this province.

To summary, healing, tissue repair and regenerative medicine is a very complicated and comprehensive science and various disciplines are involved, such as biology, developmental biology, genetics, biomaterials, tissue engineering and Chinese medicine. In the future, wound repair and regenerative medicine will be focused as follows. First, basic science plays a leading role in breaking through the bottleneck in regenerative ability in humans. Studies have shown that regenerative capacities vary greatly in the animal kingdom. Lower animals possess high regeneration capacity, but higher mammals have poor regeneration capacity. To enhance mammalian regeneration, we must understand the cellular and molecular mechanisms, such as signaling pathways and epigenetic modulation, in regeneration processes and their marked differences in animals [18, 19, 20]. Second, close cooperation between different disciplines is essential for solving the problems involved in basic science and clinical and translational application; specifically, new smart biomaterials and engineering technology play important roles in tissue regeneration, especially in artificial organ manufacture or translational applications. In 2017 and 2019, another two Xiangshan Science Conferences on Biomaterials and Regenerative Medicine organized by the academic circle highlighted the effects of new biomaterials and their application in perfect tissue repair and organ reconstruction. Finally, international cooperation is also important to push exchanges between different areas and to benefit patients as soon as possible.

## Funding

This paper was supported in part by the National Nature Science Foundation of China (81830064, 81721092) and the National Key Research and Development Plan (2017YFC1103304).

## Conflicts of interest

The author declared no potential conflicts of interest with respect to the research, authorship and/or publication of this article.
